# *PhCESA3* silencing inhibits elongation and stimulates radial expansion in petunia

**DOI:** 10.1038/srep41471

**Published:** 2017-02-02

**Authors:** Weiyuan Yang, Yuanping Cai, Li Hu, Qian Wei, Guoju Chen, Mei Bai, Hong Wu, Juanxu Liu, Yixun Yu

**Affiliations:** 1 Guangdong Key Laboratory for Innovative Development and Utilization of Forest Plant Germplasm, College of Forestry and Landscape Architecture, South China Agricultural University, Guangzhou 510642, China; 2 College of Horticulture, South China Agricultural University, Guangzhou 510642, China; 3 College of Life Sciences, South China Agricultural University, Guangzhou 510642, China

## Abstract

Cellulose synthase catalytic subunits (CESAs) play important roles in plant growth, development and disease resistance. Previous studies have shown an essential role of *Arabidopsis thaliana* CESA3 in plant growth. However, little is known about the role of CESA3 in species other than *A. thaliana*. To gain a better understanding of CESA3, the petunia (*Petunia hybrida) PhCESA3* gene was isolated, and the role of *PhCESA3* in plant growth was analyzed in a wide range of plants. *PhCESA3* mRNA was present at varying levels in tissues examined. VIGS-mediated *PhCESA3* silencing resulted in dwarfing of plant height, which was consistent with the phenotype of the *A. thaliana rsw1* mutant (a temperature-sensitive allele of *AtCESA1*), the *A. thaliana cev1* mutant (the *AtCESA3* mild mutant), and the antisense *AtCESA3* line. However, *PhCESA3* silencing led to swollen stems, pedicels, filaments, styles and epidermal hairs as well as thickened leaves and corollas, which were not observed in the *A. thaliana cev1* mutant, the *rsw1* mutant and the antisense *AtCESA3* line. Further micrographs showed that *PhCESA3* silencing reduced the length and increased the width of cells, suggesting that *PhCESA3* silencing inhibits elongation and stimulates radial expansion in petunia.

The morphology of plant organs is highly regulated through cell expansion and cell division^1^. The final shape and size of cells are often determined by the magnitude and direction of primary cell wall extension^2^,^3^,^4^. After expansion is arrested, a thick, secondary cell wall is often deposited within the boundary of the primary cell wall^5^.

In the past decades, significant insight into the molecular details of cellulose biosynthesis has been gained using forward and reverse genetic analyses. The plasma membrane rosettes contain the cellulose synthase catalytic subunit (CESA) proteins^6^,^7^,^8^,^9^,^10^. There are multiple *CESA* genes in the *CESA* gene family in plant genomes. For example, the *Arabidopsis thaliana* genome contains 10 *CESA* genes. Among these genes, *CESA1, CESA3*, and *CESA6* are required for cellulose biosynthesis in primary cell walls^11^, whereas *CESA4, CESA7*, and *CESA8* are required for cellulose biosynthesis during secondary wall deposition^6^,^7^,^8^,^9^.

In *A. thaliana*, the *CESA3* mutation (*cev1*) or antisense lines have altered plant growth, including plant height, leaf size and petal size, as well as altered fertility^12^,^13^,^14^, which are the important ornamental characteristics for ornamental plants. In addition, CESA3 is involved in resistance, activating hormone signaling and cellulose microfibril crystallinity^10^,^12^,^14^,^15^,^16^. Studies on *A. thaliana* and *Nicotiana tabacum* L. variety Samsun NN have demonstrated that the expression of the *CESA3* cellulose synthase gene that contains a point mutation, named *ixr1–2*, results in greater conversion of plant-derived cellulose to fermentable sugars^17^.

Petunia (*Petunia hybrida*) is often used as a model for studying ornamental plant growth and development. A developmental map of petunia petals has been assembled by reconstructing the pattern of cell expansion through measurement of cell size^18^.

Here, we isolated the full-length cDNA of *PhCESA3*, and its spatiotemporal expression was analyzed. VIGS-mediated *PhCESA3* silencing resulted in short but swollen stems, pedicels, filaments, styles and epidermal hairs as well as small and thickened leaves and petals. Further micrographs showed a reduced length and increased width of cells in *PhCESA3*-silenced plants. These results showed the important role of *PhCESA3* in cell elongation and radial expansion. In addition, fertility was reduced in *PhCESA3*-silenced plants.

## Results

### Isolation of *P. hybrida PhCESA3* cDNA

The full-length cDNA of *PhCESA3* was isolated from a cDNA library prepared from the corollas of petunia by homologous cloning and the RACE-PCR method. *PhCESA3* was predicted to encode a protein of 1083 amino acids with a calculated molecular weight of 120.8 kDa and a pI of 7.02.

**Figure 1:**
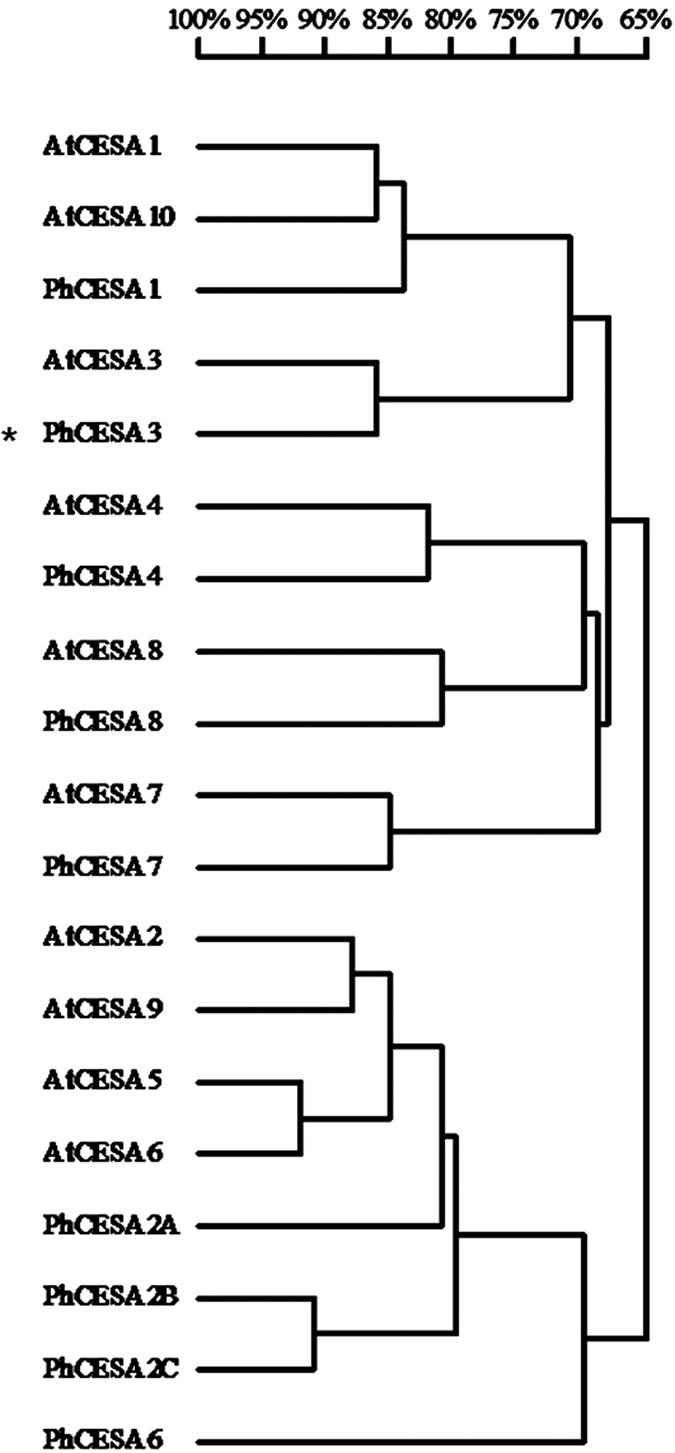
Neighbor-joining trees among proteins encoded by the *CESA*-like genes using DNAMAN. Petunia PhCESAs were aligned with *Arabidopsis thaliana* AtCESA1 (AT4G32410), AtCESA2 (AT4G39350), AtCESA3 (AT5G05170), AtCESA4 (AT5G44030), AtCESA5 (AT5G09870), AtCESA6 (AT5G64740), AtCESA7 (AT5G17420), AtCESA8 (AT4G18780), AtCESA9 (AT2G21770), and AtCESA10 (AT2G25540). Petunia PhCESAs come from Sol Genomics Network. PhCESA1, Peaxi162Scf00073g01124; PhCESA2A, Peaxi162Scf00025g00276; PhCESA2C, Peaxi162Scf00079g02433; PhCESA2B, Peaxi162Scf01294g00032; *PhCESA3*, Peaxi162Scf00953g00116; PhCESA4, Peaxi162Scf00415g00078; PhCESA6, Peaxi162Scf00041g00017; PhCESA7, Peaxi162Scf00401g00329; PhCESA8, Peaxi162Scf00371g00318.

The multiple sequence alignments of *PhCESA3* protein in the petunia and *A. thaliana* are presented in Fig. S1. *PhCESA3* shares 86.5% amino acid sequence identity with AtCESA3 (Table S1). In addition to a putative zinc-binding domain (at amino acid residues 31–76) and two putative transmembrane helices in the N-terminus, *PhCESA3* contains six putative transmembrane helices in the C-terminus (Fig. S1) similar to *A. thaliana* CESA proteins, and the C-terminus is strikingly conserved^19^,^20^. In the course of the publication of the study, the petunia genome sequences were released^21^ (https://www.sgn.cornell.edu/). By blasting against Sol Genomics Network with *A. thaliana AtCESAs*, nine *PhCESAs*, including *PhCESA3*, were obtained in the petunia genome. The *PhCESAs* are named *PhCESA1, PhCESA2A, PhCESA2B, PhCESA2C, PhCESA3, PhCESA4, PhCESA6, PhCESA7* and *PhCESA8* based on the identity of their encoding amino acid sequences with the *A. thaliana* AtCESAs (Table S1). The phylogenetic tree of the PhCESAs and *A. thaliana* AtCESAs shown in Fig. 1 further reveals that the cloned *PhCESA3* is the ortholog of AtCESA3.

### Expression patterns of *PhCESA3* mRNA

**Figure 2:**
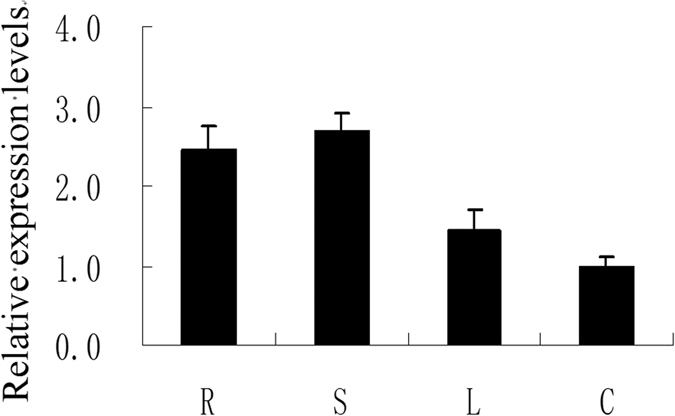
Expression of *PhCESA3* determined by quantitative real-time PCR in different organs. R, roots; L, leaves; S, stems; C, corollas. Relative expression levels are shown as fold change values. Data are presented as the mean ± SD (n = 3).

As a step toward functional analysis, we examined the spatiotemporal expression of *PhCESA3* petunia ‘Ultra’ using quantitative real-time PCR (qPCR) with gene-specific primers, and *actin* was used as an internal control. *PhCESA3* transcripts were present in root, stem, leaf, and corolla, with varying patterns of expression (Fig. 2). *PhCESA3* mRNA was slightly more abundant in roots and stems compared to corollas.

### VIGS-mediated *PhCESA3* silencing leads to plant dwarfing

**Figure 3:**
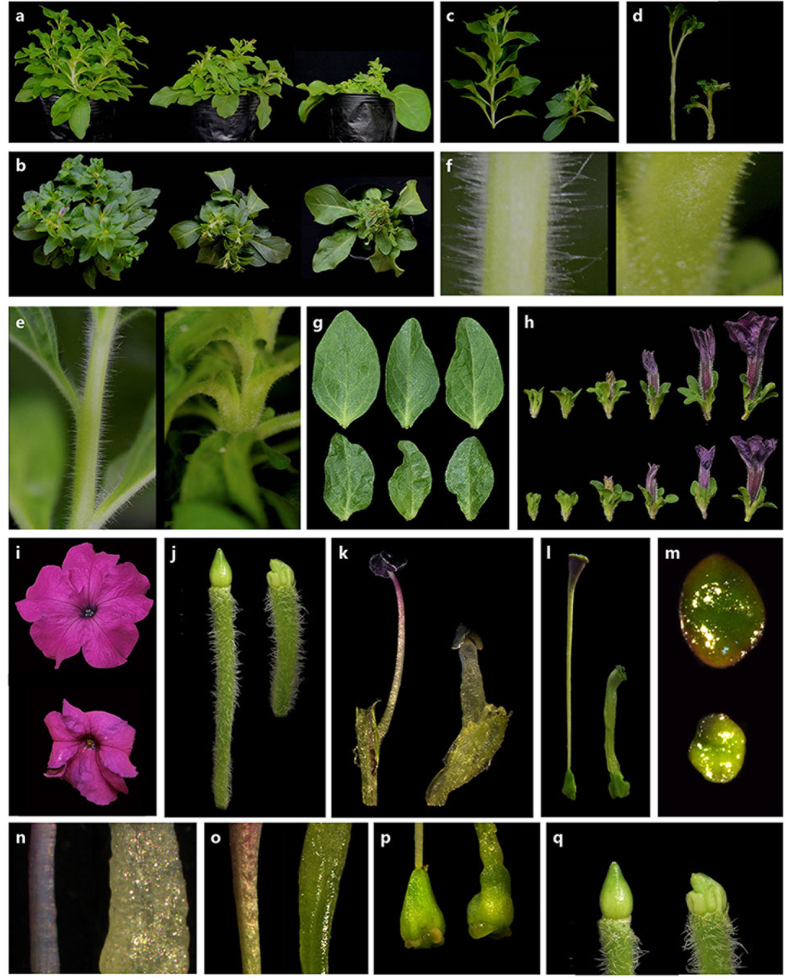
Phenotypical alteration of VIGS-mediated silencing of *PhCESA3* in plants. (**a**,**b**) Five-week-old VIGS-mediated *PhCESA3*-silenced plants (middle and right) compared to control plants (left). (**c**,**d**) Stem inflorescence of *PhCESA3*-silenced plants (right) compared with that of control plants (left) with leaves (**c**) and without leaves (**d**). (**e**,**f**) Internodes (**e**) and hairs (**f**) of stems of *PhCESA3*-silenced plants (right) compared with those of control plants (left). (**g**–**i**,**m**), Mature leaves (**g**), buds (**h**), opened flowers (**i**) and stigmas (**m**) of *PhCESA3*-silenced plants (bottom) compared with those of control plants (top). (**j**–**l**,**n**–**q**), Pedicel (**j**), filament (**k**), style (**l**), filament surface (**n**), style surface (**o**), ovary (unpollinated) (**p**) and young fruit (**q**) of *PhCESA3*-silenced plants (right) compared with those of control plants (left).

**Figure 4:**
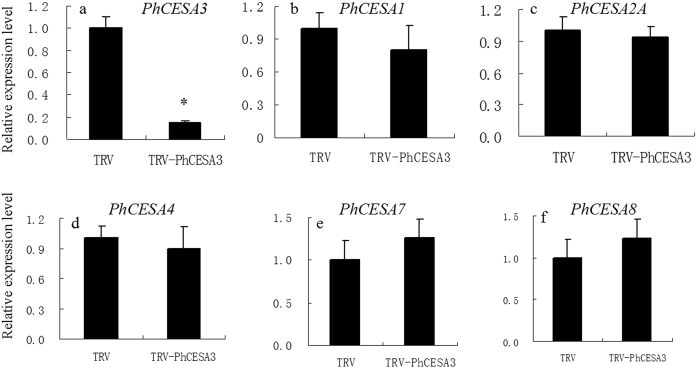
Effects of TRV2-*PhCESA3* treatment on the expression of *PhCESA3* and other *PhCESAs* in flowers on day 2 after opening as determined by quantitative real-time PCR. Relative expression levels are shown as fold change values. Data are presented as the mean ± SD (n = 3). Statistical analysis was performed using Student t test with 3 replicates. Asterisk means significant difference at P = 0. 05 level.

To explore the function of *PhCESA3*, we generated a TRV-*PhCESA3* vector that contains a 266 bp fragment of the 3′ untranslated sequence of *PhCESA3* cDNA to test the silencing of *PhCESA3* by virus-induced gene silencing (VIGS)^22^. Five weeks after TRV infection, plants infected with TRV-*PhCESA3* showed a significant dwarfing phenotype. The stems and internodes were shorter and wider than those of the empty TRV-infected control plants (Fig. 3a–e; Table 1). The silenced stems were about half the length of the control. The diameter of silenced stems was significantly increased. In addition, there were shorter stem epidermal hairs on *PhCESA3*-silenced plants compared to control plants (Fig. 3f). *PhCESA3* silencing also resulted in smaller and thicker leaves than those of control plants (Fig. 3g and Table 1). Analysis by qPCR showed decreased *PhCESA3* mRNA levels in corollas, leaves and stems in plants treated with TRV-*PhCESA3* compared to those in control plants (Figs 4 and S2 and 3).

In addition, *PhCESA3* silencing has no significantly effect on the expression of the other 8 *PhCESAs* in corollas (Fig. 4), leaves (Fig. S2) and stems (Fig. S3). *PhCESA2B, PhCESA2C*, and *PhCESA6* mRNA levels were not detected by qPCR in either TRV-*PhCESA3*-treated or control corollas and *PhCESA6* mRNA levels were not also detected in either TRV-*PhCESA3*-treated or control leaves.

### *PhCESA3* silencing alters flower organ growth

*PhCESA3* silencing reduced the height of stems but did not change the flowering time. However, altered flower organ growth was observed in *PhCESA3*-silenced plants. *PhCESA3* silencing reduced the size but increased the thickness of petal limbs, and it reduced the height of petal tubes (Fig. 3h and i; Table 1).

The pedicels (fruit stalks), filaments and styles were shorter and wider in *PhCESA3*-silenced plants than those in control plants (Fig. 3j–l; Table 1). The lengths of pedicels (fruit stalks), styles, and filaments in *PhCESA3*-silenced plants were about two-thirds, half and half of those of control plants, respectively (Table 1). The diameters of pedicels and styles of *PhCESA3*-silenced plants were about one and half, and two fold of those of control plants, respectively (Table 1). In addition, the styles and filaments were semi-transparent and loose, and their surface was rough in *PhCESA3*-silenced plants (Fig. 3n and o). In *PhCESA3*-silenced plants, the stigma was small and deformed (Fig. 3m), and the unpollinated ovaries and young fruits were often deformed (Fig. 3p and q).

### *PhCESA3* silencing leads to a significant reduction in cellulose content

**Figure 5:**
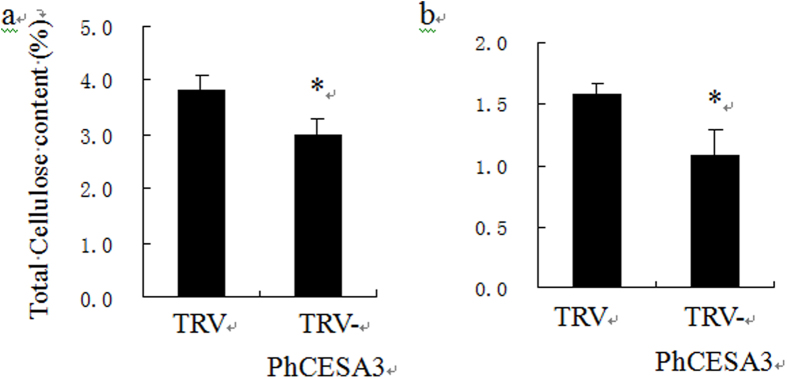
Cellulose content of mature stems and leaves from wild-type and *PhCESA3*-silenced plants. Cellulose was measured in stem segments (**a**) and leaves (**b**) from wild-type and *PhCESA3*-silenced plants. Standard error bars are shown (n = 6). Statistical analysis was performed using Student t test with 6 replicates. Asterisk means significant difference at P = 0.05 level.

To further characterize the function of *PhCESA3*, cellulose content was analyzed in plants treated with TRV and TRV-*PhCESA3* treatment plant. *PhCESA3*-silenced plants showed a significant decrease in total cellulose content of the stems and leaves (Fig. 5a and b). In 5-week-old plants, mature stems in *PhCESA3*-silenced plants had about 80% and mature leaves had about 70% of the control level of cellulose (Fig. 5a and b).

### *PhCESA3* silencing leads to a significant reduction in fertility

The effects of *PhCESA3* silencing on fertility were examined. *PhCESA3* silencing caused a significant reduction in fertility, resulting in fewer than 100 seeds per fruit. More seeds per fruit were obtained in reciprocal crosses between *PhCESA3*-silenced plants and control plants when *PhCESA3*-silenced plants were used as the pollen donor or receptor (Table 2) but fewer than crosses between both control plants as parents, indicating reduced female and male fertility. Reciprocal crosses with control plants showed that the female reproductive capacity was more strongly affected than male reproductive capacity in *PhCESA3*-silenced plants (Table 2).

### *PhCESA3* silencing inhibits cell elongation and causes cell swelling

**Figure 6:**
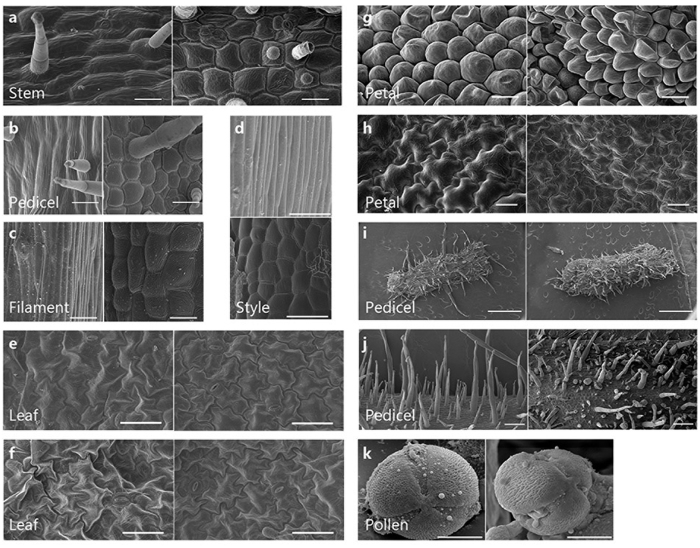
Scanning electron micrographs of the *PhCESA3*-silenced plants compared with those of control plants. (**a**–**d**), Epidermal cells of stem (**a**), pedicel (**b**), filament (**c**) and style (**d**) showing reduced cell length and increased cell width in *PhCESA3*-silenced plants (right, bottom in (**d**)) compared to control plants (left, top in (**d**)). (**e**,**f**), Adaxial (**e**) and abaxial (**f**) epidermal cells of leaves showing the reduced cell size in *PhCESA3*-silenced plants (right) compared to control plants (left). (**g**,**h**) Adaxial (**g**) and abaxial (**h**) epidermal cells of petal limb showing the reduced cell size in *PhCESA3*-silenced plants (right) compared to control plants (left). (**i**), Pedicels showing the reduced number of trichomes in *PhCESA3*-silenced plants (right) compared to control plants (left). (**j**) Trichomes of pedicel showing the reduced trichome length and more collapsed trichomes in *PhCESA3*-silenced plants (right) compared to control plants (left). (**k**) Pollen grains showing the abnormal pollen mother cell in *PhCESA3*-silenced plants (right) compared to control plants (left). Bars = 50 μm in (**a**–**f**); bars = 20 μm in (**g**–**h**); bar = 1 mm in (**i**); bars = 200 μm in (**j**); bars = 10 μm in (**k**).

Because *PhCESA3* silencing altered the growth of stems, leaves and flowers, we further examined these organs in detail. Scanning electron micrographs showed reduced cell length and increased cell width of the epidermal cells of stems, pedicels, filaments and styles in *PhCESA3*-silenced plants (Fig. 6a–d; Table 3). Optical micrographs of the epidermis of stems and pedicels showed similar results (Fig. S4a and b).

**Figure 7:**
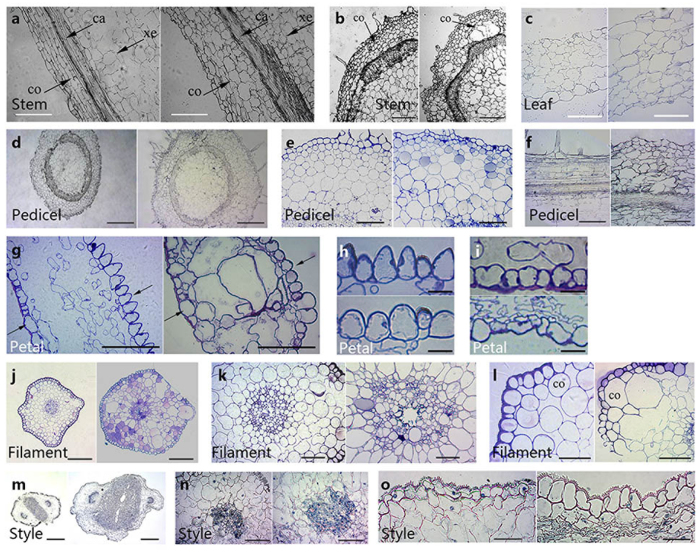
Section micrographs of *PhCESA3*-silenced plants compared to control plants. (**a**) Stem longitudinal section showing the increased width of cortex and cortical cells in *PhCESA3*-silenced plants (right) compared to control plants (left). (**b**) Stem transverse section showing the uneven surface in *PhCESA3*-silenced plants (right) compared to control plants (left). (**c**) Leaf transverse section showing the thickened leaf in the *PhCESA3*-silenced plants (right) compared to the control plant (left). (**d**) Pedicel transverse section showing the thickened pedicel in the *PhCESA3*-silenced plants (right) compared to the control plant (left). (**e**,**f**) Pedicel transverse (**e**) and longitudinal (**f**) sections showing the increased width of cortical cells in *PhCESA3*-silenced plants (right) compared to control plants (left). (**g**) Petal limb transverse section showing the thickened petal limb in the *PhCESA3*-silenced plants (right) compared to the control plant (left). Solid and hollow arrows indicate the cell shape of the adaxial and abaxial sides, respectively. (**h**) The adaxial epidermal cells of petal limb showing sleek cells in the *PhCESA3*-silenced plants (bottom) compared to the control plant (top). (**i**) The abaxial epidermal cells of petal limb showing the uneven size and irregular shape of cells in the *PhCESA3*-silenced plants (bottom) compared to the control plant (top). (**j**,**m**) Transverse section showing the thickened filament (**j**) and style (**m**) in the *PhCESA3*-silenced plants (right) compared to the control plant (left). (**k**,**n**) Marrow transverse section of filament and style showing the irregularly arranged marrow cells and different cell sizes in the *PhCESA3*-silenced plants (right) compared to the control plant (left). (**l**) Filament cortex transverse section showing the irregularly arranged cortical cells and uneven cell sizes in the *PhCESA3*-silenced plants (right) compared to the control plant (left). (**o**), Style transverse section showing the width of epidermal cells in the *PhCESA3*-silenced plants (right) compared to the wild-type plant (left). The cortex (co), cambium (ca), and xylem elements (xe) are indicated. Bars = 250 μm in (**a**,**b**,**f**,**j**,**m**); bars = 100 μm in (**e**,**c**); bars = 500 μm in (**d**,**l**); bars = 50 μm in (**g**,**k**,**n**,**o**); bars = 10 μm in (**h**,**i**).

As visualized in transverse and longitudinal sections, *PhCESA3* silencing also reduced the length and increased the width of the cortical cells of stems, pedicels, and filaments, but the number of cortical cells did not change (Fig. 7a,b,e,f and l; Table 3), indicating that the swollen stems were attributed to an increased cell width rather than a decrease in cell number. Hand-cut sections also showed similar results (Fig. S5a and b). The transverse sections showed wider pedicels, filaments and styles in *PhCESA3*-silenced plants than those in control plants (Fig. 7d,j,m; Table 1). In addition, the arrangement of cortical cells in stems, pedicels and filaments was irregular and close in *PhCESA3*-silenced plants compared with that of control plants (Fig. 7b,e,l). The cortical cells were often round or oval shaped in control plants, while the cortical cells were often irregular polygon shaped in *PhCESA3*-silenced plants (Fig. 7b,e and l).

Scanning electron micrographs of adaxial and abaxial epidermal cells of leaves and petal limbs showed reduced cell size in *PhCESA3*-silenced plants (Fig. 6e and h). The reduced proportion of leaves and petal limbs was similar to that of the epidermal cells of leaves and petal limbs. Optical micrographs of the epidermis of leaves and petals showed similar results (Fig. S4c–f). As visualized in transverse sections, *PhCESA3* silencing increased the width of the mesophyll cells of leaves and petals (Fig. 7c and g), while the number of mesophyll cells did not change, indicating that the thickened leaves and petals were attributed to an increased cell size rather than cell number.

**Figure 8:**
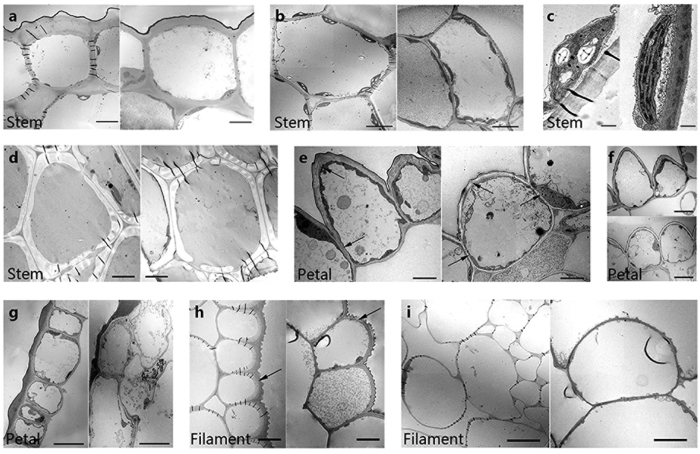
Transmission electron micrographs of *PhCESA3*-silenced plants compared to control plants. Mature inflorescence stems were cross-sectioned for imaging of interfascicular fiber cell walls. (**a**) Stem epidermal cells showing the reduced thickness of cell wall in the *PhCESA3*-silenced plants (right) compared to the control plant (left). (**b**) Stem cortical cells showing similar cell wall thickness in *PhCESA3*-silenced plants (right) and wild-type plants (left). (**c**) Chloroplast of stem cortical cells showing the reduced starch granules in the *PhCESA3*-silenced plants (right) compared to control plants (left). (**d**) Stem xylem cells showing similar cell wall thickness in *PhCESA3*-silenced plants (right) and control plants (left). (**e**) Petal limb epidermal cells showing the reduced cell wall thickness in *PhCESA3*-silenced plants (right) compared to control plants (left). (**f**) Petal limb upper epidermal cells showing the change of cell shape in *PhCESA3*-silenced plants (bottom) compared to control plants (top). (**g**) Petal limb lower epidermal cells showing the change of cell shape and reduced cell wall thickness in *PhCESA3*-silenced plants (right) compared to control plants (left). (**h**) Filament epidermal cells showing the change of cell shape, cell size and reduced cell wall thickness in *PhCESA3*-silenced plants (right) compared to control plants (left). (**i**) Filament cortical cells showing changed cell shape and size but similar cell wall thickness in *PhCESA3*-silenced plants (right) compared to control plants (left). Bars = 10 μm in (**a**,**b**,**f**–**i**); bars = 1 μm in (**c**); bars = 2.5 μm in (**d**); bars = 5 μm in (**e**).

In addition, the sections of petal limbs revealed that the outside shape of the epidermal cells of the adaxial side of petal limbs from control plants was often sharp, while the outside shape of the same cells from *PhCESA3*-silenced plants was curved (Fig. 7g and h). The epidermal cells of the abaxial side of petal limbs of control plants showed a similar size and good arrangement, but the same cells in *PhCESA3*-silenced plants had different sizes, different shapes, and irregular arrangement (Fig. 7g and i). Transmission electron micrographs of cells from petal limbs confirmed these results (Fig. 8e–g).

Scanning electron micrographs showed that the epidermal cells of petals in *PhCESA3*-silenced plants were often collapsed while those of control plants were plump (Fig. 6g). The collapsed cells may have been due to thin and fragile epidermal cell walls in *PhCESA3*-silenced plants compared to those of control plants as revealed by transmission electron microscopy (Fig. 8e).

The transverse sections of filaments and styles showed the wide marrow in *PhCESA3*-silenced plants compared with that of the control (Fig. 7k and n). Moreover, the myeloid cells of control plants were similar in size and had a good arrangement while those of *PhCESA3*-silenced plants had different sizes, different shapes and irregular arrangement (Fig. 7k and n). In addition, wider epidermal cells of style were observed in the *PhCESA3*-silenced plants compared to those in the control plants (Fig. 7o).

Scanning electron and optical micrographs of hand-cut sections and stereomicroscope micrographs all showed that *PhCESA3* silencing reduced the length and increased the width of cells of epidermal hairs in stems and pedicels (Fig. S5c and d). Scanning electron microscopy showed the increase of number of epidermal hairs of pedicels in the *PhCESA3*-silenced plants (Fig. 6i). In addition, scanning electron micrographs showed collapsed epidermal hairs on *PhCESA3*-silenced plants, which may be attributed to the thin and fragile cell walls in *PhCESA3*-silenced plants (Fig. 6j).

In addition, scanning electron micrographs of pollen grains showed that parts of the pollen cell wall were collapsed in *PhCESA3*-silenced plants, but the pollen cells in control plants were plump (Fig. 6k), which may partially explain the sterility of *PhCESA3*-silenced plants.

### *PhCESA3* silencing alters the ultrastructure of cells

To investigate if *PhCESA3* silencing affects the ultrastructure of cells, we examined the thickness of cell walls. Transmission electron micrographs of cell walls revealed that the epidermal cell walls of stems, petal limbs and filaments in *PhCESA3*-silenced plants were thin compared to those of control plants (Fig. 8a,e,f,g and h; Table 4), while the thickness of the cell walls of cortical cells and xylem cells of stems was not significantly different between the plants (Fig. 8b and d; Table 4). In addition, we observed that the starch granules in chloroplasts of stem cortical cells in *PhCESA3*-silenced plants were rare compared to control plants (Fig. 8c).

## Discussion

*CESA3*, which encodes the catalytic subunit 3 of cellulose synthase, has been shown to be involved in the biosynthesis of primary cell walls in *A. thaliana*^12^,^13^,^14^,^15^. We isolated a petunia homolog of the *A. thaliana CESA3* gene (*PhCESA3*), and we analyzed the spatial and temporal regulation of *PhCESA3* expression. Furthermore, we produced VIGS-mediated *PhCESA3*-silenced plants and compared these plants to control plants to examine the role of *PhCESA3* in plant growth and development.

The predicted peptide sequence of *PhCESA3* shows high similarity to *A. thaliana* CESAs throughout the protein with a putative zinc-binding domain and two putative transmembrane helices in the N-terminus as well as six putative transmembrane helices in the C-terminus (Fig. S1). *PhCESA3* has the homology with AtCESA3 in *A. thaliana*, which indicates that *PhCESA3* is the homolog of AtCESA3. *PhCESA3* mRNA was detected in all organs examined but was present at different levels in different organs (Fig. 2), indicating that *PhCESA3* expression is spatially regulated.

In the flowers of plants treated with TRV-*PhCESA3, PhCESA3* mRNA levels showed decreased compared to those of control flowers, while the mRNA levels of other *PhCESAs* were not significantly changed compared to those of control flowers (Fig. 4), which showed the specific silencing of *PhCESA3* in plants treated with TRV-*PhCESA3*.

In *A. thaliana*, mutants of *AtCESA4, 7*, and *8* specifically show a cellulose defect in the secondary wall of the xylem^15^,^23^,^24^, whereas *AtCESA1, 3* and *6* are mainly involved in cellulose synthesis in the primary cell wall^5^,^25^,^26^. AtCESA1 and AtCESA3 are required for cellulose synthesis in the primary cell wall, whereas AtCESA2 and AtCESA6 may be at least partially redundant^9^. Null alleles of *AtCESA3* and *AtCESA1* are embryo lethal^27^,^28^. In *A. thaliana*, the *rsw1* mutant (the temperature-sensitive allele of *AtCESA1*) the antisense *AtCESA3* line, and the *cev1* mutant (a mild mutation of *AtCESA3*) results in dwarfing of plants, small leaves, small petals, short pedicels, short filaments, short styles, and reduced fertility compared to control plants^12^,^13^,^14^,^15^. The short stamen filament surface of the *rsw1* mutant is crumpled^29^. In tobacco, using the VIGS-method silencing of *NtCESA1*, the homolog of *AtCESA3*, leads to shorter internode lengths, small leaves, and a dwarf phenotype^30^. In this study, similar results were obtained in the *PhCESA3*-silenced plants (Fig. 3a–e,g,i–l). Both the antisense *AtCESA3* line and *rsw1* mutant affect female reproductive competence more severely than male competence in *A. thaliana*^13^,^29^, which is consistent with the results in this study (Table 2). However, *PhCESA3* silencing caused swollen stems, pedicels, filaments, styles and epidermal hairs, which were not observed in the *At*CESA3 antisense line, the *cev1* mutant and the *rsw1* mutant in *A. thaliana*^12^,^13^,^14^,^15^,^29^. Further, scanning, transmission and optical micrographs showed that *PhCESA3* silencing caused short and swollen cells of these organs or tissues, which was consistent with the corresponding phenotypes. Thus, *PhCESA3* silencing resulted in primary cell wall extension in length and width. Importantly, growing the *rsw1* mutant at its restrictive temperature inhibits root and hypocotyl elongation as well as promotes radial swelling^29^. Mutations in *A. thaliana AtCESA3* and *AtCESA6* confer resistance to isoxaben (an herbicide that specifically inhibits cellulose) and isoxaben, which causes radial swelling of roots^15^. Taken together, with strong inhibition of cellulose synthesis, whether genetic or chemical (i.e., with herbicides such as isoxaben), organ elongation is inhibited and radial expansion is stimulated. However, the organs that exhibit radial expansion induced by *CESA* silencing are different in various species.

Among the ten *AtCESAs* in *A. thaliana, AtCESA3 (IXR1*/*CEV1*) is suggested to be essential for depositing cellulose in primary walls^10^. When changes to primary walls alter cell expansion and/or cell divisions, changes to cell shape, growth, and morphogenesis occur. Changes to thickening after growth stops occur too late to affect these processes, resulting in normal morphology but changes in mechanical properties^13^. Similar to the *A. thaliana AtCESA3* mutant, *PhCESA3* silencing resulted in significant changes in the cell size and shape in several organs. Moreover, the cellulose content of mature stems and leaves from *PhCESA3*-silenced plants was significantly decreased compared to that of control plants. These results suggested that *PhCESA3* mainly functions for catalyzing the biosynthesis of cellulose deposited to the primary wall. Ultrastructural studies clearly showed that the cell walls of the epidermis in stems and petals, which are heavily thickened during primary growth^31^, were thinner, thus further supporting the involvement of *PhCESA3* in depositing cellulose in the primary wall. The thickness of the cell walls of cortical cells and xylem cells of stems did not significantly change (Fig. 8b and d; Table 4), suggesting that *PhCESA3* is not involved in depositing cellulose in thickening walls after cell growth stops.

In this study, the shorter stems, pedicels, filaments and styles in *PhCESA3*-silenced plants were attributed to reduction in cell length rather than cell number, and these swollen organs in *PhCESA3*-silenced plants were attributed to increased cell width rather than cell number (Fig. 6a–d; Fig. 7a,b,e,f,j and l). These results implicated that *PhCESA3* is mainly involved in cell elongation rather than cell division, which is consistent with results obtained with the antisense *AtCESA3* line in *A. thaliana*^12^,^13^,^14^,^15^. In addition, plant organ shaping requires control over cell wall expansion anisotropy, which is characterized by the direction and degree of anisotropy^32^. In this study, the inhibited elongation and stimulated radial expansion of several organs in *PhCESA3*-silenced plants showed the change of the direction and degree of anisotropy of cell wall.

Here, *PhCESA3* silencing resulted in changes of plant height, flower size and fertility, which are the important features of ornamental plants. These results suggested the important role of *PhCESA3* in controlling plant stature and form. By using specific promoters, changes in *CESA3* expression in different organs may create an excellent variety for ornamental plants. Therefore, *PhCESA3* may be utilized in petunia breeding for changing the plant height, flower size and fertility.

## Methods

### Plant material

Petunia ‘Ultra’ plants were grown under greenhouse conditions (22–25 °C, 14 h light/10 h dark). Flowers were emasculated 1 d before the flowers were fully open to prevent self-pollination. Eight to ten petunia flowers were harvested at anthesis (corollas 90° reflexed) stages and were then placed immediately in tap water. Stem, leaves and roots were collected from plants at the vegetative stage when the plants were approximately 10 cm in height. All tissues were frozen in liquid nitrogen and stored at −80 °C until used for RNA extraction. Fresh weights were measured immediately before freezing. All experiments were conducted at least three times with independently collected and extracted tissues unless otherwise noted.

### RNA extraction, RT-PCR and cloning of the petunia *PhCESA3* gene

Total RNA was extracted and reverse-transcribed according to the methods of Liu *et al*.^33^. The partial sequences of *PhCESA3* were obtained using a computational identification approach^34^. Briefly, TBLASTN analysis against the GenBank EST database (http://www.ncbi.nlm.nih.gov) with *A. thaliana CESA3* identified 1 petunia clone, FN016492, which encodes putative proteins that displayed conservation with *A. thaliana* CESA3. The remaining 5′ and 3′ cDNA sequences of *PhCESA3* were isolated by RACE using a specific primer (Table S2), and its full-length cDNA was isolated by RT-PCR.

### Sequence analysis

Alignments were performed, and a phylogenetic tree was generated using the DNAMAN software. Identity search for nucleotides and translated amino acids was performed using the National Center for Biotechnology Information (NCBI) BLAST network server (http://www.ncbi.nlm.gov/BLAST).

### Quantitative real-time PCR assays

Quantitative real-time PCR (qPCR) assays were performed according to previous methods^33^. Analyses were conducted following the Minimum Information for Publication of Quantitative Real-Time PCR Experiments guidelines^35^. Petunia *Actin* (accession no. FN014209) and *Cyclophilin (CYP*) (accession no. EST883944) genes were used as the internal reference genes to quantify the cDNA abundance^36^. Similar results were obtained for both reference genes. The data presented in the body of the text represent relative expression values calculated using *Actin*, and the data presented in the attachment represent relative expression values calculated using *CYP*. The sequences of all primers used for qPCR analysis are described in Table S3.

### Agroinoculation of TRV vectors

To generate pTRV2 containing the 3′ untranslated region of *PhCESA3* (TRV2-*PhCESA3*), the gene sequence of 266 bp was PCR amplified using forward primers and reverse primers (Table S4), and the PCR products were inserted into the pTRV2 vector. *Agrobacterium tumefaciens* (strain GV3101) transformed with pTRV1 and pTRV2 derivatives were prepared as previously described^22^,^37^. The *Agrobacterium* cells grown overnight were harvested and resuspended in inoculation buffer containing 10 mM MES, 200 mM acetosyringone, and 10 mM MgCl_2_ to an OD_550_ of 10. Following an additional 3 h of incubation at 28 °C, bacteria transformed with pTRV1 were mixed with bacteria containing the pTRV2 derivatives in a 1:1 ratio, and 200 to 400 μL of this mixture was injected into the stem or applied on the cut surface after removing the apical meristems of petunia plantlets. Approximately thirty plants were vaccinated with each vector. The inoculated plants were grown under greenhouse conditions (22–25 °C, 14 h light/10 h dark).

### Cellulose measurement

Cellulose content in mature stems and leaves was measured by a previously published method^38^,^39^. Analysis was conducted with material from a single plant using three to five fully expanded leaves (from 5-week-old plants) or a 3 cm segment from the base of the primary inflorescence stem. Stems were chopped with a razor blade, and a crude cell wall fraction was obtained by extracting the soluble material with two changes of 70% ethanol at 70 °C for 1 h each^40^. The ethanol was removed, and the samples were dried under vacuum. The dry weight of the wall material was recorded. Cellulose was measured according to a previously published method^39^.

### Scanning electron micrographs

The stems, leaves, pedicels, petal limbs, filaments, styles and anthers from *PhCESA3*-silenced plants and control plants were cut into 3–5 mm^2^ pieces. The samples were fixed in 4% glutaraldehyde in 0.1 mol/L PBS (pH 7.2) for 4 h at 4 °C and then washed 3 times in the same buffer, which was followed by post-fixation in 1% osmium tetroxide for 2 h at room temperature and 3 rinses using the same buffer. Samples were dehydrated in increasing grades of ethanol and then dried with a critical point drier (CPD 030, Switzerland, Bal-Tec). The dried samples were fixed on the sample stage and coated with gold by ion sputtering equipment. Samples were observed with a scanning electron microscope (XL-30-ESEM, The Netherlands, FEI) at 10 kV acceleration and photographed.

### Paraffin sections

The stems, leaves, pedicels and styles from plants were cut into 5 mm × 5 mm × 5 mm pieces. The samples were fixed in FAA fixative solution (every 100 ml of FAA fixative solution contains 90 ml of 50% or 70% ethanol, 5 ml of acetic acid, and of 5 ml formalin) for 24 h at room temperature and then washed in running water for 24 h. Samples were stained by hematoxylin for 4 d and then washed in running water for 24 h, which was followed by dehydration in increasing grades of ethanol. A graded chloroform series was used for clearing, and the samples were embedded in paraffin. Paraffin sections were cut to a thickness of 8 μm on a Leica RM2235 followed by dewaxing with xylene. Lastly, slides were sealed with neutral resin. Sections were observed and photographed with a Zeiss Scope.A1 microscope.

### Semi-thin sections

The pedicels, petal limbs and filaments from *PhCESA3*-silenced plants and control plants were cut into 1 mm × 1 mm × 0.5 mm pieces. The specimens were fixed in 2.5% paraformaldehyde/3.0% glutaraldehyde in 0.1 mol/L PBS (pH 7.2) for 4 h at 4 °C, and the specimens were then washed 3 times in the same buffer, which was followed by post-fixation in 1% osmium tetroxide for 2 h at room temperature and 3 rinses using the same buffer. The specimens were dehydrated in a graded ethanol series and embedded in Epon812 (SPI Supplies Division of Structure Probe Inc., West Chester, PA, USA). Polymerization took place for 24 h at 40 °C, which was followed by 24 h at 60 °C. Specimens were cut to a thickness of 1 μm on a Leica RM2155 and were stained with 0.5% toluidine blue. Sections were observed and photographed with a Leica DMLB microscope.

### Transmission electron micrograph

The stems, petal limbs and filaments from *PhCESA3*-silenced plants and control plants were cut into <1 mm^3^ pieces. The samples were fixed in 4% glutaraldehyde in 0.1 mol/L PBS (pH 7.2) for 4 h at 4 °C and then washed 3 times in the same buffer, which was followed by post-fixation in 1% osmium tetroxide for 2 h at room temperature and 3 rinses using the same buffer. Samples were dehydrated in increasing grades of ethanol and embedded in Epon812 (containing 51.64% Epon812, 5.37% DDSA, 42.99% MNA and 1.5% DMP-30). Polymerization took place for 24 h at 45 °C, which was followed by 24 h at 60 °C. Ultrathin sections (100 nm thick) were cut with a Leica ULTACUT ultramicrotome and deposited on a 200 mesh copper net with support film. Samples were stained with a 2% uranyl acetate solution followed by a 6% lead citrate solution, and samples were visualized with a Tecnai 12 transmission electron microscope (FEI, Eindhoven, The Netherlands) at 80 kV acceleration and photographed.

## Additional Information

**How to cite this article:** Yang, W. *et al. PhCESA3* silencing inhibits elongation and stimulates radial expansion in petunia. *Sci. Rep.* **7**, 41471; doi: 10.1038/srep41471 (2017).

**Publisher's note:** Springer Nature remains neutral with regard to jurisdictional claims in published maps and institutional affiliations.

## Supplementary Material

Supplemental Tables and Figures

## Figures and Tables

**Table 1:** *PhCESA3* silencing alters plant growth.

	TRV (Control)	TRV-*PhCESA3*	TRV-*PhCESA3*/TRV (%)
Height of plant (cm)	18.75 ± 1.28	10.65 ± 2.03*	57
Length of internode (cm)	2.8 ± 0.58	0.68 ± 0.20*	24
Diameter of stem (mm)	2.25 ± 0.21	3.73 ± 0.52*	166
Length of leaf (cm)	4.45 ± 0.58	3.32 ± 0.23*	75
Width of leaf (cm)	2.46 ± 0.31	1.84 ± 0.26*	75
Thickness of leaf (μm)	198.67 ± 32.55	337.93 ± 65.96*	170
Length of pedicel (cm)	3.88 ± 0.9	1.15 ± 0.28*	30
Diameter of pedicel (mm)	1.82 ± 0.21	2.75 ± 0.36*	151
Diameter of corolla (cm)	6.71 ± 0.48	3.81 ± 0.65*	57
Thickness of petal limb (μm)	103.24 ± 12.91	160.92 ± 33.81*	156
Length of filament (cm)	1.19 ± 0.54	0.62 ± 0.17*	52
Diameter of filament (mm)	0.44 ± 0.03	0.80 ± 0.17*	182
Length of style (cm)	2.16 ± 0.08	1.30 ± 0.21*	60
Diameter of style (mm)	0.62 ± 0.07	1.20 ± 0.24*	194
Thickness of stem cortex (μm)	174.72 ± 24.43	301.69 ± 37.68*	173
Thickness of pedicels cortex (μm)	277.22 ± 33.74	359.73 ± 60.44*	130

^*^Data are means ± SE from 15 to 20 samples. Statistical analysis was performed using Student t test with 15 to 20 replicates. Asterisk means significant difference at P = 0. 05 level.

**Table 2:** Effects of *PhCESA3* silencing on the fertility.

	TRV self-fertilization	TRV-*PhCESA3* self-fertilization	TRV ♀ and TRV-*PhCESA3*♂	TRV-*PhCESA3*♀ and TRV ♂
Number of seeds per fruit	205 ± 13a	92 ± 11d	155 ± 12b	118 ± 12c

^*^Data are means ± SE from 15 to 20 fruits. Different letters mean significant difference at P = 0. 05 level.

Statistical analysis was performed using one way analysis of variance (ANOVA) followed by Duncan’s multiple range test (DMRT) with 15 to 20 replicates.

*P*-values ≤0.05 were considered as significant.

**Table 3:** Effects of *PhCESA3* silencing on the cell size

	TRV (Control)	TRV-*PhCESA3*	TRV-*PhCESA3*/TRV (%)
Height of epidermal cells of stem (μm)	89.69 ± 14.17	50.74 ± 14.59*	57
Width of epidermal cells of stem (μm)	26.50 ± 5.56	55.47 ± 14.29*	209
Height of cortex cells of stem (μm)	117.07 ± 19.06	67.83 ± 16.42*	58
Width of cortex cells of stem (μm)	28.29 ± 4.76	65.67 ± 12.37*	232
Diameter of adaxial epidermal cells of leaf (μm)	101.52 ± 6.23	84.59 ± 7.86*	83
Diameter of abaxial epidermal cells of leaf (μm)	93.39 ± 5.67	76.67 ± 6.46*	82
Height of epidermal cells of pedicel (μm)	166.09 ± 56.89	66.76 ± 18.63*	40
Width of epidermal cells of pedicel (μm)	32.50 ± 3.71	40.51 ± 3.37*	125
Height of cortex cells of pedicel (μm)	161.02 ± 20.30	83.41 ± 33.94*	52
Width of cortex cells of pedicel (μm)	58.76 ± 9.38	117.77 ± 20.56*	200
Diameter of abaxial epidermal cells of petal limb (μm)	72.28 ± 8.62	48.73 ± 7.32*	67
Height of epidermal cells of filament (μm)	63.53 ± 7.67	25.78 ± 4.60*	41
Width of epidermal cells of filament (μm)	18.88 ± 3.20	30.99 ± 6.02*	164
Width of cortex cells of filament (μm)	29.46 ± 2.84	76.94 ± 14.44*	261
Height of epidermal cells of style (μm)	119.07 ± 14.13	62.74 ± 14.91*	53
Width of epidermal cells of style (μm)	16.78 ± 2.81	26.60 ± 5.62*	159
Width of cortex cells of style (μm)	29.15 ± 7.34	41.86 ± 9.40*	144

^*^Data are means ± SE from 15 to 20 samples. Statistical analysis was performed using Student t test with 15 to 20 replicates. Asterisk means significant difference at P = 0. 05 level.

**Table 4:** Effects of *PhCESA3* silencing on the cell walls.

	TRV (Control)	TRV-*PhCESA3*	TRV-*PhCESA3*/TRV (%)
Epidermal cell walls of stems (μm)	5.35 ± 0.60	4.38 ± 0.44*	82
Adaxial epidermal cell walls of petal limbs (μm)	1.71 ± 0.54	0.92 ± 0.02*	54
Abaxial epidermal cell walls of petal limbs (μm)	2.33 ± 0.55	0.59 ± 0.03*	25
Epidermal cell walls of filaments (μm)	2.84 ± 0.21	1.71 ± 0.16*	60
Cortical cell walls of stems (μm)	1.27 ± 0.08	1.18 ± 0.08*	93
Xylem cell walls of stems (μm)	1.16 ± 0.06	1.19 ± 0.07*	103

^*^Data are means ± SE from 15 to 20 samples. Statistical analysis was performed using Student t test with 15 to 20 replicates. Asterisk means significant difference at P = 0. 05 level.
