# Supplementary material for: PhCESA3 silencing inhibits elongation and stimulates radial expansion in petunia
Source: Sci Rep. 2017 Feb 2;7:41471. doi: 10.1038/srep41471 (PMC5288708; doi:10.1038/srep41471)
Supplement: Supplemental Tables and Figures [file srep41471-s1.doc]

**Title: *PhCESA3* silencing inhibits elongation and stimulates radial expansion in petunia**

Weiyuan Yang^1, 2^, Yuanping Cai^1^, Li Hu^1^, Qian Wei^1^, Guoju Chen^2^, Mei Bai^1, 3^, Hong Wu^1, 3^, Juanxu Liu^1^, Yixun Yu^1^

1 Guangdong Key Laboratory for Innovative Development and Utilization of Forest Plant Germplasm, College of Forestry and Landscape Architecture, South China Agricultural University, Guangzhou 510642, China

2 College of Horticulture, South China Agricultural University*,* Guangzhou 510642, China

**Table S1** Comparative analysis of PhCESAs amino acid sequences with *Arabidopsis* *thaliana* AtCESAs

| Identity (%) | PhCESA1 | PhCESA2A | PhCESA2B | PhCESA2C | **PhCESA3** | PhCESA4 | PhCESA6 | PhCESA7 | PhCESA8 |
| --- | --- | --- | --- | --- | --- | --- | --- | --- | --- |
| AtCESA1 | **85.80** | 64.70 | 66.00 | 64.80 | 70.90 | 66.20 | 64.70 | 68.30 | 68.40 |
| AtCESA2 | 66.50 | **81.60** | **80.30** | **81.10** | 65.80 | 65.50 | 70.00 | 68.40 | 63.70 |
| AtCESA3 | 73.30 | 65.50 | 65.00 | 64.80 | **86.50** | 68.50 | 66.50 | 70.90 | 69.40 |
| AtCESA4 | 67.60 | 64.40 | 63.70 | 63.40 | 69.00 | **82.30** | 63.20 | 67.50 | 70.90 |
| AtCESA5 | 66.90 | 81.30 | 78.50 | 79.10 | 67.20 | 66.20 | 69.50 | 67.30 | 64.50 |
| AtCESA6 | 66.10 | 80.90 | 79.40 | 80.00 | 66.00 | 65.50 | **70.10** | 67.40 | 64.20 |
| AtCESA7 | 69.00 | 67.10 | 65.60 | 65.20 | 71.40 | 69.20 | 67.10 | **85.30** | 69.40 |
| AtCESA8 | 66.60 | 63.90 | 63.90 | 62.10 | 66.30 | 68.00 | 64.20 | 66.90 | **81.30** |
| AtCESA9 | 66.10 | 79.90 | 78.10 | 77.90 | 64.60 | 65.20 | 68.70 | 66.80 | 64.50 |
| AtCESA10 | 81.30 | 63.20 | 63.60 | 62.40 | 69.10 | 64.30 | 63.20 | 66.60 | 66.90 |

**Table S2** Specific primer sequences of ***PhCESA3*** used in for cloning gene

| Forward primer (5′→3′) | Reverse primer (5′→3′) |
| --- | --- |
| CAGATCGTCTGAACCAAGTGC | CCAAATCTCTTTTCCAGGCTC |

**Table S3** Primer sequences of ***PhCESAs*,** *Phactin* **and** *PhCYP* used in quantitative real-time PCR

| Gene | Forward primer (5′→3′) | Reverse primer (5′→3′) |
| --- | --- | --- |
| ***PhCESA3*** | TTGTTATGGGTGCGGATTG | TCATTTGCTGCATGGTTG |
| *PhCESA1* | TCAGAAGCCACGAAGACT | GAGGGAAGCAATGACAGA |
| *PhCESA2A* | TATTCATCTTTACCCATTCC | TTGACCCGTACCCACAAC |
| *PhCESA2B* | AATGCTTTGATGCGAGTG | TTTCTTTCCTGAAGTGGG |
| *PhCESA2C* | ATCCAGGGTCCAATTTAC | CTTTCTTATGCTTCTTTCG |
| *PhCESA4* | AGGGACAAATCCAACAAC | CTCCGTGACAGAACCATA |
| *PhCESA6* | TGCTCACTAATGCTCCCT | CAATGCCATCAAACCTCT |
| *PhCESA7* | TTTGGCTGGCATAGATAC | TTGTTGGAGGAATAAGGA |
| *PhCESA8* | TGGCTGTGGTTATGAAGA | CTCAATGGCATACAGTAAAT |
| *PhActin* | TGCTGATCGAATGAGCAAGGAA | GGAGCAACAACCTTAATCTTC |
| *PhCYP* | AGGCTCATCATTCCACCGTGT | TCATCTGCGAACTTAGCACCG |

**Table S4** Primer sequences of ***PhCESA3*** used in VIGS

| Gene | Forward primer (5′→3′) | Reverse primer (5′→3′) |
| --- | --- | --- |
| ***PhCESA3*** | GCGGATCCATTGCTAGAAAAACTCAAGT | CGGAATTCAAAGCATTAAGGACACTGTA |

<===========================VR1============

=================================>

<======================VR2=====================

=>

★★★★★

V

V

V

**Figure S1** Alignment of the Amino Acid Sequences of Several Plant Cellulose Synthase Genes. Petunia PhCESA3 were aligned with *Arabidopsis thaliana* AtCESA1 (AT4G32410), AtCESA3 (AT5G05170), AtCESA7 (AT5G17420) and AtCESA10 (AT2G25540). Solid boxes indicate regions in which more than half of the residues are identical; gray boxes indicate conserved residues. The positions of three aspartic acid (D) residues and QxxRW motifs are indicated by vertical arrowheads and asterisks, respectively. Variable regions VR1 and VR2 are also indicated. Dashes were introduced to optimize alignment.

Relative expression level

Relative expression level

*PhCESA3*

*PhCESA1*

*PhCESA2A*

*PhCESA2B*

*PhCESA2C*

*PhCESA4*

*PhCESA7*

*PhCESA8*

Relative expression level

*

b

a

c

e

d

f

g

h

**Figure S2** Effects of TRV2-PhCESA3 treatment on the expression of *PhCESA3* and other *PhCESAs* in mature leaves as determined by quantitative real-time PCR. Relative expression levels are shown as fold change values. Data are presented as the mean ± SD (n = 3). Statistical analysis was performed using Student t test with 3 replicates. Asterisk means significant difference at P=0. 05 level.

Relative expression level

Relative expression level

*PhCESA3*

*PhCESA1*

*PhCESA2A*

*PhCESA2B*

*PhCESA2C*

*PhCESA4*

*PhCESA6*

*PhCESA7*

Relative expression level

*PhCESA8*

*

b

a

c

d

e

f

g

h

i

**Figure S3** Effects of TRV2-PhCESA3 treatment on the expression of *PhCESA3* and other *PhCESAs* in stems 2 cm below the apex flowers on day 2 after opening as determined by quantitative real-time PCR. Relative expression levels are shown as fold change values. Data are presented as the mean ± SD (n = 3). Statistical analysis was performed using Student t test with 3 replicates. Asterisk means significant difference at P=0. 05 level.


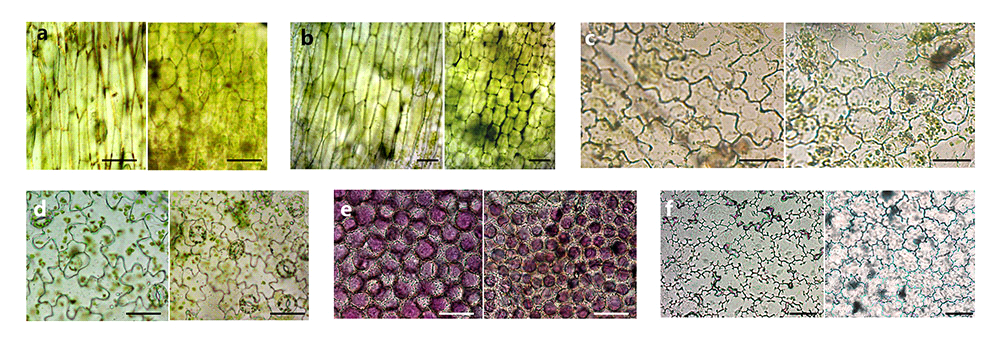


**Figure S4** Epidermal cells micrographs of *PhCESA3*-silenced plants compared to wild-type plants. (a) and (b), Epidermal cells of stem (a) and pedicel (b) showing the reduced cell length and increased cell width in *PhCESA3*-silenced plants (right) compared to control plants (left). (c) and (d), Adaxial (c) and abaxial (d) epidermal cells of leaves showing the reduced cell size in *PhCESA3*-silenced plants (right) compared to control plants (left). (e) and (f), Adaxial (e) and abaxial (f) epidermal cells of petal limbs showing the reduced cell size in *PhCESA3*-silenced plants (right) compared to control plants (left). Bars=50 μm.


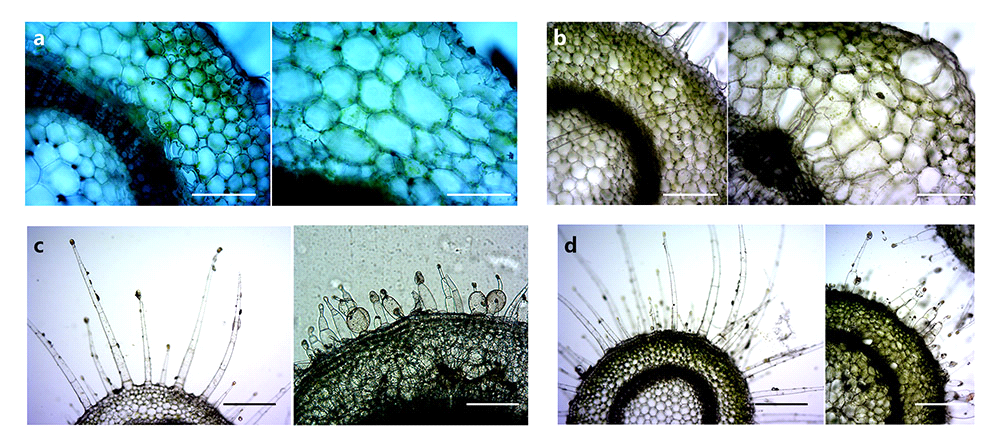


**Figure S5** Micrographs of hand-cut sections of *PhCESA3*-silenced plants compared to control plants. (a) and (b), Transverse section of stem (a) and pedicel (b) showing the increased width of cortical cells in *PhCESA3*-silenced plants (right) compared to control plants (left). (c) and (d), Transverse section showing the reduced length and increased width of hair cells of the stem (c) and pedicel (d) in *PhCESA3*-silenced plants (right) compared to control plants (Left). Bars=100 μm in (a); bars=200 μm in (b); bars=500 μm in (c) and (d).
